# [Corrigendum] 3-Bromopyruvate and sodium citrate target glycolysis, suppress survivin, and induce mitochondrial-mediated apoptosis in gastric cancer cells and inhibit gastric orthotopic transplantation tumor growth

**DOI:** 10.3892/or.2026.9096

**Published:** 2026-03-11

**Authors:** Ting-An Wang, Xiao-Dong Zhang, Xing-Yu Guo, Shu-Lin Xian, Yun-Fei Lu

Oncol Rep 35: 1287–1296, 2016; DOI: 10.3892/or.2015.4511

Subsequently to the publication of the above paper, an interested reader drew to the Editor's attention that, regarding the transmission electron microscopy experiments shown in Fig. 9 on p. 1295, the two ‘sCt’ panels on the left in the lower row, and the ‘5-Fu’ panel the furthest on the right in the top row and the ‘sCt’ panel second on the right in the lower row, contained overlapping sections of data, albeit these areas were at different sizes and/or in different orientations.

The authors were able to re-examine their original data files, and realized that the ‘5-Fu’ panel the furthest on the right in the top row had inadverently been selected incorrectly (the two abovementioned ‘sCt’ panels on the left in the lower row were correctly placed, showing the different fields of view). The revised version of Fig. 9, now featuring the correct data for the ‘5-Fu’ panel in the top row, is shown below. Note that the error made in assembling this figure did not affect the overall conclusions reported in the paper. The authors are grateful to the Editor of *Oncology Reports* for allowing them the opportunity to publish this Corrigendum, and apologize to the readership for any inconvenience caused.

## Figures and Tables

**Figure 9.**
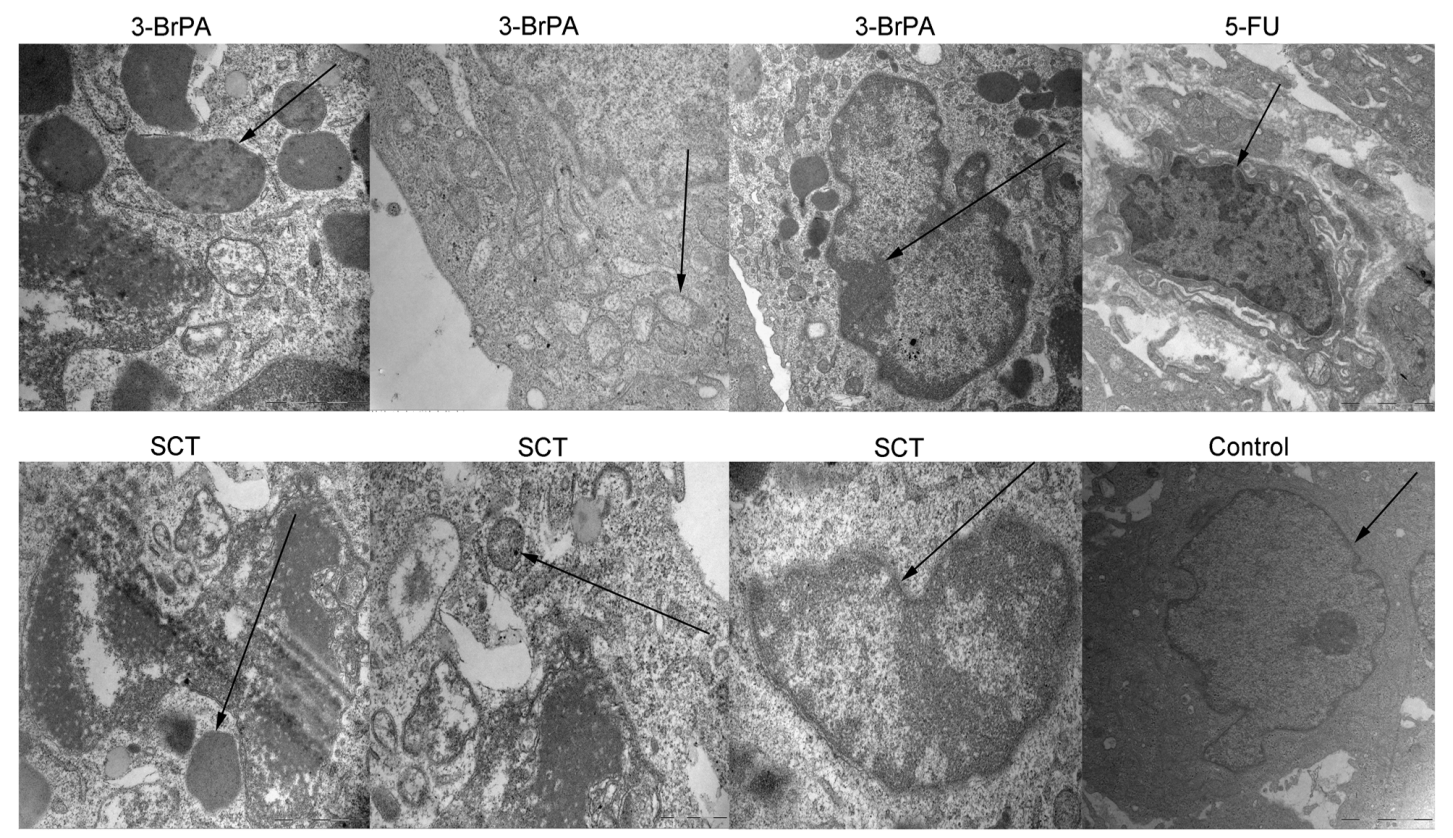
3-BrPA and SCT mediate changes in tumor ultrastructure as observed by TEM. Typical features of apoptosis such as formation of apoptotic bodies, mitochondrial crest fracture or disappearance and chromatin concentration or fragmentation were observed in the 3-BrPA, SCT or 5-FU groups. However, the PBS-treated group had large nuclei with prominent nucleoli and no apoptotic bodies were observed.

